# The rubber manufacturing industry: a case report and review of cutaneous exposure and sequelae

**DOI:** 10.1186/s12995-015-0075-4

**Published:** 2015-09-04

**Authors:** Claire Powers, Heather P. Lampel

**Affiliations:** School of Medicine, Duke University, Durham, North Carolina USA; Department of Dermatology, Duke University, Durham, North Carolina USA

## Abstract

Exposure to chemical carcinogens in rubber manufacturing remains a serious occupational health concern. Workers are exposed to these carcinogens via skin or inhalation. Rubber manufacturing work is associated with a high prevalence of dermatologic diseases such as eczema, allergic contact dermatitis and atopic dermatitis. The role that epidermal exposure plays in the development of malignancies historically associated with the rubber industry is less certain. We present a case relevant to this discussion and review the role of skin exposure in the rubber industry, providing an overview of the cutaneous and systemic manifestations of occupational exposures in modern day rubber workers.

## Background

In their seminal report in 1982, the International Agency for Research on Cancer (IARC) determined the rubber industry “entailed exposures that are carcinogenic to humans [1].” During the following decades, control measures aimed at reducing exposure were instituted, such as removal of known carcinogens from the rubber production process. As a result, exposure to airborne particulate [2] and dermal exposure in rubber factories [3] has decreased drastically since the 1980s.

Rubber production has increased considerably in Asia over the past century, with fewer factories in Europe and North America. In 1989, 54,600 workers were employed in the United States rubber industry, compared to 88,300 workers in 1977 [4]. However, the majority of health data regarding the rubber industry arises from Western countries [5].

The process of rubber manufacturing is complex and continually changing, thus making the study of occupational exposures in the rubber industry difficult. Rubber workers’ route and compounds of chemical exposure depend largely on the specific employee work task and environment. In 1994, the Centers for Disease Control and the National Institute for Occupational Safety and Health summarized these various exposures. Handling raw materials can give rise to considerable amounts of airborne dust particulate, while mixing, extruding, calendaring and vulcanizing (curing) rubber exposes workers to inhaled fumes. The specific carcinogens contained in these fumes have not yet been fully identified, yet studies have demonstrated the mutagenic potential of the fumes [6, 7]. Vulcanizing rubber releases N-nitrosamines and polycyclic aromatic hydrocarbons, both known carcinogens. Workers in final inspection and finishing tasks have extensive skin contact with finished rubber [4].

## Case report: a tire-maker’s traumatic tattoos

**Fig. 1:**
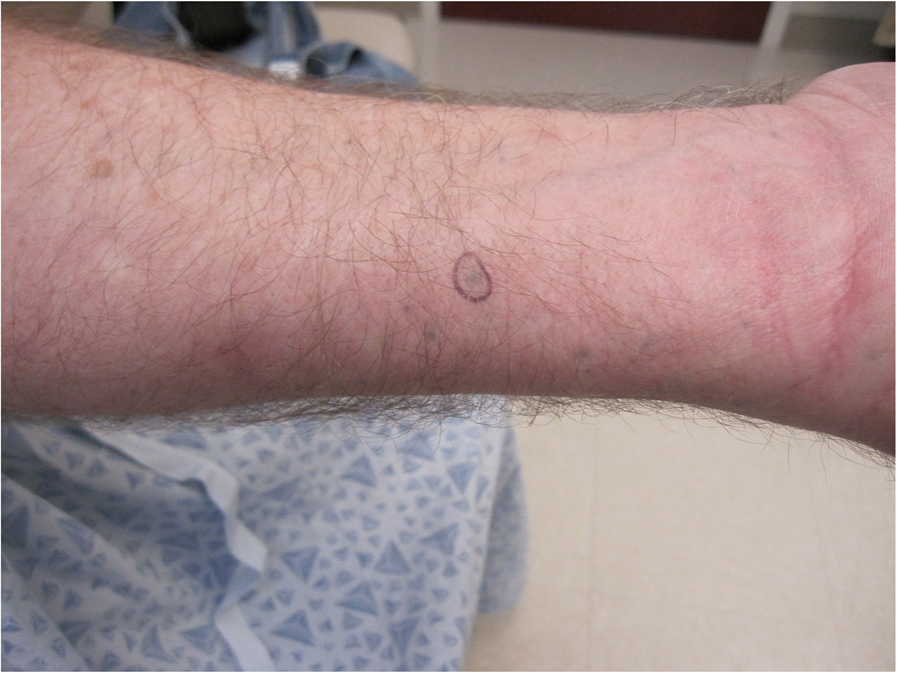
Forearm demonstrating numerous blue-black macules

**Fig. 2:**
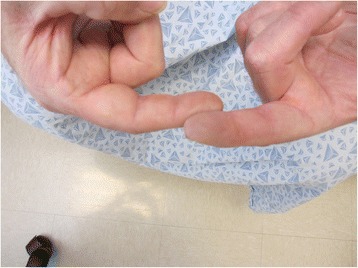
Coalescent hyperpigmentation of the distal fingertip due to traumatic rubber tattoos

A 57-year-old white male patient who has worked in a tire manufacturing plant for 37 years and retired 6 years prior, presented with asymptomatic dermally-implanted rubber in the bilateral hands, forearms and upper arms (Fig. 1). The dermally implanted rubber is most pronounced on his left index finger, which is darkly discolored (Fig. 2). These “rubber tattoos” resulted from frequent trauma while cutting unvulcanized rubber implanted with sharp metal strips. His work task was specifically manipulating and hand cutting unvulcanized rubber sheets which had uniform, thin metal wires embedded. These metal wires also extended beyond the ends of the sheets, exposing the worker to repeated puncture wounds. He reported regular notice of rubber particulate on his hands. It is likely that these repeated wire punctures essentially “tattooed” the worker’s skin over time with rubber particles by traumatic implantation. He wore forearm sleeves for over a decade, but not gloves. The patient noted that many coworkers in his department had similar skin findings. Biopsy of these lesions demonstrated perivascular pigment consistent with rubber tattoo. No systemic biomarkers were measured given his lengthy retirement. The patient expressed an understanding of the well-known link between cancer and the rubber manufacturing industry [8] but was specifically inquisitive about the increased risk of malignancies given his dermally implanted “rubber tattoos.”

## Skin exposure

Manufacturing jobs in general harbor the largest burden of occupational skin diseases compared with other industries [9] and dermal exposure is an important route of toxin acquisition, especially in the rubber industry. Repetitive skin contact with warm unfinished rubber products is thought to be the primary route for dermal contamination [10]. Depending on the factory conditions and the use of protective equipment, soluble dermal exposure may be as much as tenfold higher than inhaled amounts [3]. This may not be the case in curing departments, where exposure to large quantities of cyclohexane-soluble fumes occurs [10]. Soluble cyclohexane was adapted by the British Rubber Manufacturers association as a measure of the fraction of the fume or dust that “contains the constituents most likely to be harmful” to human health [11]. Indeed, one study of 225 Dutch rubber tire workers in 2008 demonstrated that increased levels of soluble dermal cyclohexane on the hands and wrists was correlated with increased urinary levels of polycyclic aromatic hydrocarbons but that urinary mutagenicity seemed to be more associated with inhalable dust exposure [12]. However, other studies have observed a direct relation between urinary mutagenicity and skin contamination, notably after controlling for cigarette use [13, 14]. Therefore, intake through the skin may play an important role in occupational exposure to mutagenic compounds in the rubber industry.

## Cutaneous manifestations of occupational exposure in the rubber industry

### Dermatitis

Frequent causes of allergic contact dermatitis, rubber additives such as accelerators, antioxidants and vulcanizers are increasingly utilized in the modern manufacturing process to improve quality, speed of production, and product durability [15, 16]. Multiple chemicals have been identified as sensitizing agents for rubber-related contact dermatitis (Table 1) [4]. The prevalence of type IV hypersensitivity reactions to rubber constituents is estimated between 3.8 % and 4.4 % [15, 17]. These allergies are often related to rubber glove use in health services and laboratory workers; however, a distinct subset of allergies due to non-glove, industrial exposure in rubber manufacturers exists. A cross-sectional survey of 999 workers in an Australian tire plant reported a prevalence rate of 37 cases of occupational contact dermatitis per 1,000 workers, highlighting the risks of dermatitis associated with the rubber industry [18].

**Table 1:** Agents reported to cause contact dermatitis in rubber product workers according to the CDC & NIOSH 1993 report (Table adapted from Reference 4)

Chemical	CAS Number	Process	Product
2-(2′-4′ dinitrophenylthio) benzothiazole	17586–89–9	All areas	Tires
4,4′-dithiodimorpholine, 1 %	103–34–4	Not specified	Tires
n-isopropyl-n- phenylparaphenylenediamine (IPPD)	101–72–4	Assembly, maintenance, compounding	Tires
n-dimethyl-1,3 butyl-n- phenylparaphenylenediamine	793–24–8	Assembly, maintenance, compounding	Tires
para-phenylenediamine compounds	106–50–3	Handling of uncured rubber	Tires, footwear
ethylene thiourea (ETU)	96–45–7	Sewing	Non-tire
resorcinol	108–46–3	Not specified	Tires

### Skin cancer

The relationship between occupational rubber exposure and skin cancer is less certain. Increased incidence of cutaneous squamous cell carcinoma among workers exposed to rubber stock and lubricating oils was found in a nested case–control study in Akron, Ohio in 1987 [19]. The distribution of these cancers on the body was similar to a sun-exposed population, with the head and neck most common, followed by the hands and arms. This unexpected distribution questions whether these cancers were indeed related to an occupational exposure. Exposure to well—accepted skin carcinogens polycyclic aromatic hydrocarbons (PAHs) [1] is greatest in the mixing, milling, and assembly areas where direct handling of uncured rubber stock occurs [19]. PAHs are still very much present in the modern day rubber industry, as demonstrated by measuring higher weekday vs. weekend urinary 1-hydroxypyrene levels (a valid marker for PAH exposure) in rubber workers in 2008 [12]. The IARC acknowledged an increased risk of skin cancer in 1982, also noting that lubricating oils in uncured rubber contain known skin carcinogens [1]. However, a more recent Swedish study conducted in 2007 examined 5,745 rubber workers and failed to demonstrate increased risk of skin cancer in the rubber industry [20]. In 2012, the IARC concluded that there is insufficient evidence to determine the relationship between the rubber industry and skin cancer [5].

## Cancers other than skin

In an updated 2012 report, the IARC reviewed three-decades of new data on the rubber industry. The IARC re-confirmed there is sufficient evidence of excess risk of bladder, leukemia, lung and stomach cancer in the modern rubber manufacturing industry. This recent report differed from the previous 1982 report in that the IARC acknowledged an excess risk of malignant lymphomas including multiple myeloma and esophageal cancers in rubber industry workers, while finding inconclusive evidence to link laryngeal cancer to the rubber industry. Prostate, brain, thyroid and pancreatic cancer continue to have insufficient evidence of increased risk in the rubber industry [5].

Well-documented associations of cancer in the rubber industry related to specific occupational exposures include the excess risk of leukemia with benzene exposure [20, 21], bladder cancer with 2-naphthylamine [22] and o-toluidine exposure [23], lung cancer with asbestos and carbon black [24], and nitrosamines released during vulcanization posing excess risk of pharyngeal, esophageal and cancers of the oral cavity [25]. This distinction highlights the importance of understanding worker duties and exposures when advising industrial rubber workers about cancer risk. A prospective cohort study from 1999 on 11,633 German rubber workers conducted in five different plant departments found the highest mortality in the early production stages involving mixing and weighing of uncured rubber products [24]. They hypothesized that mixing and weighing disturbs large quantities of dusts, possibly talc, asbestos or carbon black, while directly handling uncured rubber exposes to PAHs. In our case study, we report the patient worked directly with unvulcanized rubber in these early production processes, which was noted by this study of German rubber workers to be of high risk. Specific biomarkers exist for monitoring some chemical exposures (Table 2).

**Table 2:** Accepted biomarkers of exposure in the rubber industry

Chemical	Biomarker
Asbestos	Lung x-rays and pulmonary function testing; others in development
Polycyclic aromatic hydrocarbons	Urine 1-hydroxypyrene
Benzene	Urine s-phenylmercapturic acid; urine t,t-muconic acid

## Conclusion

Our patient worked in a tire-manufacturing plant for more than three decades. His specific role in the manufacturing line contributed to skin exposure to unvulcanized rubber stock. He also suffered repeated puncturing of the skin from the wires in his medium and subsequent “rubber tattoos.” No studies to date address the subsequent health risks from skin-implanted rubber. Our patient did not have any current evidence of allergic contact dermatitis and no history of cancers. As mentioned above, rubber workers in early production stages working with uncured rubber and those with direct, extensive skin-contact with rubber precursors may have higher rates of bladder, leukemia, lung, stomach cancers, malignant lymphomas and esophageal cancers. In alignment with the IARC, we find that there is insufficient evidence to support increased risk of skin cancer in such workers. However, rubber stock is known to contain polycyclic aromatic hydrocarbons, demonstrated skin-carcinogens that have not been eliminated from the modern rubbery industry. Therefore, evidence suggests that rubber workers with extensive skin contact, especially direct, repetitive traumatic introduction of uncured rubber into the skin may be a unique sub-group in this industry deserving further study.

It is wise to recommend personal protective equipment including gloves to prevent skin contact with uncured rubber in such employment. However, our particular patient stated that any glove use compromised his tactile ability. He did wear forearm sleeves for some of his employment, and these can be considered. In areas of fumes or dust particulate, we recommend task-specific mask filters for inhalation prevention. Enclosing processes and automating tasks when possible could decrease worker exposure.

Further research is needed to determine the risks of skin cancer in the dynamic rubber industry, as additional study could directly support safer handling practices and the further removal of carcinogens from the industry for workers.

## Consent

Written informed consent was obtained from the patient for publication of this Case report and any accompanying images. A copy of the written consent is available for review by the Editor-in-Chief of this journal.

## Abbreviations

(IARC): International Agency for Research on Cancer
(PAH): Polycyclic aromatic hydrocarbon
